# Global burden of athletic-type knee dislocation in young adults: a GBD 2021 proxy-based analysis, 1990–2021

**DOI:** 10.3389/fphys.2025.1692607

**Published:** 2026-01-06

**Authors:** Tariq Alkhatatbeh, Ahmad Alkhatatbeh, Weidong Chen, Hang Fang, Yan Liao, Jingyang Cheng, Hao Liang, Zhilin Liang, Hao Chen, Wenyan Huang, Zijie Fang, Rongkai Zhang

**Affiliations:** 1 Department of Joint Surgery, Center for Orthopaedic Surgery, The Third Affiliated Hospital of Southern Medical University (Academy of Orthopedics Guangdong Province), Guangzhou, Guangdong, China; 2 Orthopedic Hospital of Guangdong Province, Guangzhou, Guangdong, China; 3 Guangdong Provincial Key Laboratory of Bone and Joint Degeneration Diseases, Guangzhou, Guangdong, China; 4 Department of Orthopedics, The First Affiliated Hospital of Shantou University Medical College, Shantou, Guangdong, China

**Keywords:** global burden of disease, incidence, joinpoint, knee dislocation, prevalence, sports injuries, uncertainty intervals, young adults

## Abstract

**Background:**

Knee dislocation is an uncommon but limb-threatening injury that often arises during athletic activities and can result in neurovascular compromise, multi-ligament disruption, and long-term disability. However, the Global Burden of Disease (GBD) framework does not include a specific sports-injury category, so the contribution of athletic-type mechanisms to the global burden of knee dislocation in young adults remains unclear. We, therefore, used GBD 2021 data to estimate the young-adult component of the global burden of knee dislocation from 1990 to 2021, describe long-term trends, and compare a proxy for athletic-type mechanisms with other external causes.

**Methods:**

We performed a secondary analysis of Global Burden of Disease 2021 estimates for knee dislocation, extracting annual incidence and prevalence for adults aged 20–40 years between 1990 and 2021 by sex. A proxy for athletic-type mechanisms was defined as cases assigned to the external-cause categories “falls” and “other exposure to mechanical forces” and was compared with transport injuries and all other external causes. We calculated population age-standardized rates per 100,000 populations with 95% uncertainty intervals and summarized temporal patterns using average annual percentage change derived from log-linear regression models.

**Results:**

Absolute incidence counts increased for all causes (1023103→1084122; +5.96%) and the athletic proxy (577923→675111; +16.82%), increasing the proxy’s share of 20–40 incidence from 56.49% to 62.27%. Population-weighted incidence rates decreased for all causes (61.18→46.12 per 100000; −24.62%, APC −0.94%) and the proxy (34.56→28.72; −16.90%, APC −0.74%). Prevalence rates also decreased (all causes 11.20→8.59; proxy 6.12→5.08), while prevalence counts increased (all causes 187256→202052; proxy 102281→119503). In 2021, proxy rates were higher in male individuals than in female individuals (incidence 36.49 vs. 20.76; prevalence 6.06 vs. 4.08 per 100,000). Within the proxy, falls increased from 76.58% to 81.12% of incident cases.

**Conclusion:**

Young adults carry a large global burden of knee dislocation, consistent with athletic trauma. Despite increasing counts, population-weighted rates fell steadily from 1990 to 2021; male individuals remain at higher risks, and falls dominate the proxy mechanism. The proxy approach offers a reproducible way to monitor athletic-type knee dislocations where sports-injury labels are absent.

## 1 Introduction

Knee dislocation, although uncommon at the population level, is a serious injury that carries a disproportionate risk of neurovascular injury, multi-ligamentous disruption, and long-term functional loss (Seroyer et al., 2008; Chowdhry et al., 2020). Recent epidemiological studies distinguish between isolated knee dislocations and those occurring in multi-trauma patients, noting distinct injury patterns and outcomes that require specialized management strategies (Darcy et al., 2018). Population summaries emphasize early vascular and nerve complications, with one meta-analysis finding vascular injury in 10.7% of cases (Constantinescu et al., 2023). However, the long-term burden extends beyond acute management; Moatshe et al. (2017a) and Moatshe et al. (2017b) reported that despite surgical intervention, osteoarthritis prevalence can reach 46% within a decade, underscoring the potential for persistent disability . This makes systematic prevention and surveillance a priority for public health.

Yet, routine reporting often undercounts or misclassifies events as the incidence of knee dislocation is often underestimated due to spontaneous or easily assisted reductions (Yang et al., 2011). Furthermore, most data systems do not distinguish athletic mechanisms from other external causes, which limits inference about sports-related risks in young adults (Arom et al., 2014). Despite the clinical importance of knee dislocations, current surveillance systems face critical limitations. Athletic trauma represents a distinct mechanism with younger age at injury and specific force patterns compared to motor vehicle accidents or low-energy falls. However, the International Classification of Diseases does not systematically capture activity context, making it impossible to directly quantify sports-related burden from routine data. Without reliable estimates, sports governing bodies lack baseline metrics to evaluate rule changes or equipment standards, public health agencies cannot allocate prevention resources efficiently, and clinicians miss opportunities to target high-risk athletic populations.

Recent global analyses using the Global Burden of Disease (GBD) (Institute For Health Metrics And Evaluation, 2024) framework have mapped the burden of various injuries and disorders (Vos et al., 2020; Wu et al., 2021). Complementing these findings, Cieza et al. (2020) have quantified the substantial global need for rehabilitation services arising from musculoskeletal conditions, reinforcing the public health relevance of these injuries. This massive undertaking systematically examines diseases and risk factors across hundreds of countries to help researchers and policymakers understand global health trends. Previous work identified falls and high-energy trauma among the leading causes of knee dislocation. These findings anchor the international context but do not isolate athletic trauma and predate the latest GBD data cycle.

Chen et al. (2024) used GBD 2019 to quantify global knee-dislocation burden from 1990 to 2019, reporting incidence and years lived with disability across regions and age–sex groups, with increasing counts, decreasing age-standardized rates, a male excess, and falls as the leading cause. Their analysis did not isolate athletic mechanisms and predated the 2021 cycle. Beyond knee dislocations specifically, musculoskeletal disorders have become a leading cause of disability among adolescents and young adults worldwide, with particularly rapid increases in high-SDI settings (Guan et al., 2023). Complementing these population-level patterns, a recent systematic review of 43,869 knee dislocations reported substantial neurovascular complications, persistent functional limitations, and low rates of return to sport and work, underscoring the long-term burden in this predominantly young-adult population (Randall et al., 2024). We, therefore, update to GBD 2021 and explicitly separate the most consistent portion with athletic trauma using a transparent proxy. Given the increasing global participation in organized sports among young adults (Hulteen et al., 2017), we hypothesized that although absolute case counts would increase due to population growth, the risk (incidence rate) attributable to athletic mechanisms might show distinct trends compared to transport injuries due to evolving safety standards.

This study addresses this gap. We focus on ages 20–40, the period of peak athletic exposure and a high-risk demographic for this injury, particularly for young adult male individuals. The objective of this study is to estimate the global burden of knee dislocation in young adults (aged 20–40) from 1990 to 2021, particularly isolating the “athletic proxy” component. We quantify the proxy share of incidence and prevalence, compare sex-specific risks, and describe evolving cause patterns. This provides a reproducible, policy-relevant estimate of the athletic component that the current GBD structure does not report explicitly.

## 2 Methods

### 2.1 Data sources and study population

We used Global Burden of Disease 2021 estimates (IHME GBD results tool) at the global level for calendar years 1990–2021. The analytic population comprised four five-year age bands (20–24, 25–29, 30–34, and 35–39), aggregated to represent ages 20–40. Both the combined series and the sex-specific series for male and female individuals were examined. Inclusion comprised all global GBD 2021 records for ages 20–40 (both sexes) the study causes, and measures; exclusion comprised records outside this age range or below the global level and observations with missing values in the source. For each year, age band, sex, and cause, we extracted incidence and prevalence counts and used matching global population denominators aligned to the same age–sex structure to construct rates per 100,000.

Data were extracted via the IHME GBD Results tool (https://vizhub.healthdata.org/gbd-results) in August 2025. All processing used R 4.5.2 with tidyverse for data manipulation and ggplot2 for visualization. No primary data collection occurred; all analyses used publicly available aggregate estimates, requiring no institutional review board approval.

### 2.2 Case definition and outcome measures

Because the GBD cause list does not provide a sports-injury category, we defined a pre-specified proxy for athletic knee dislocation as the sum of two external causes in the GBD hierarchy: falls and other exposure to mechanical forces. “Falls” (W00–W19) encompasses slipping, tripping, and stumbling, which are consistent with non-contact ligamentous mechanisms common in sports (e.g., ACL rupture leading to dislocation). “Other exposure to mechanical forces” (W20–W64) includes being struck by objects or persons, consistent with contact-sport mechanisms. Transport injuries were analyzed in parallel as a comparator, an external cause less directly linked to athletic participation. All-cause series served as the reference for reporting the proxy share in adults aged 20–40. Inclusion criteria were as follows: 1. Global GBD 2021 estimates; 2. age groups 20–24, 25–29, 30–34, and 35–39 years; 3. calendar years 1990–2021; and 4. measures of incidence and prevalence. Exclusion criteria were as follows: 1. data from regions below the global level (to maintain statistical power for the proxy); 2. age groups outside the 20–40 range.

For each measure, counts were summed across the four age bands to generate a total for ages 20–40 for every year. We derived structural descriptors that included the proportion of the proxy attributable to falls versus mechanical forces, the distribution of cases across the four age bands within ages 20–40, male-to-female ratios and absolute gaps, and the prevalence-to-incidence ratio computed within the same year and cause set. Where all-ages series were available, the proportion of the global burden occurring in individuals aged 20–40 was calculated as the ratio of the 20–40 total to the all-ages total for that year.

### 2.3 Rate construction and statistical analysis

To obtain interpretable rates for ages 20–40, we merged age-specific case counts with the matching age-specific population denominators by year and sex and then summed cases and population across the four bands. For year t and sex s, the rate per 100,000 equals 100,000 multiplied by the ratio of the summed cases to the summed population across 20–24, 25–29, 30–34, and 35–39. This procedure was applied to all causes, to each proxy component, and to the combined proxy, for both sexes combined and male and female individuals separately.

Long-run changes were summarized with log-linear time-trend models fit to annual series. For a series observed yearly, we modeled the natural log of the rate as a linear function of time and reported the annual percent change as 100 × [exp (slope)−1]. A single candidate change in slope was evaluated by scanning a breakpoint across admissible years and accepted when the Bayesian information criterion improved by more than 10 relative to a single-segment model. When a joinpoint was accepted, slopes and annual percent changes were refit within each segment.

Uncertainty was incorporated where GBD provided lower and upper bounds. For each year and cause, age-band counts for the lower and upper bounds were summed across the four bands, and rates per 100,000 were then computed using the summed population denominators. This aggregation yields conservative uncertainty ribbons because it does not model cross-band correlation. We displayed 95% uncertainty intervals for historical rates and performed a sensitivity analysis by re-fitting the trend model to the historical lower and upper series.

Forecasts to 2050 were generated by extrapolating the last-segment log-linear slope on the log scale and transforming back to the rate scale. Ninety-five percent prediction intervals were derived from the residual variance of the fitted model on the log scale. We report central forecasts and prediction intervals for 2030, 2040, and 2050. Forecasts are presented for rates; conversion to counts would require corresponding population projections and is outside the scope of the main analysis.

## 3 Results

### 3.1 Global trends in incidence and prevalence

From 1990 to 2021, the global knee-dislocation burden in ages 20–40 increased in absolute numbers but decreased when expressed as population-weighted rates. As detailed in Table 1, all-cause incident cases increased by 5.96% (from 1,023,103 to 1,084,122). However, the athletic proxy (falls + mechanical forces) increased significantly faster (+16.82%), reaching 675,111 cases in 2021. Consequently, the proxy’s share of total incidence increased from 56.49% to 62.27%.

**TABLE 1:** Endpoint counts and rates (1990 vs. 2021, ages 20–40, both sexes).

**Category**	**1990 count**	**2021 count**	**Absolute change**	**% change**	**1990 rate**	**2021 rate**	**Proxy share**
**All causes (Inc)**	1023103	1084122	+61019	+5.96%	61.18	46.12	n/a
**Proxy (Inc)**	577923	675111	+97188	+16.82%	34.56	28.72	56.49%→62.27%
**Falls (Inc)**	442518	547631	+105113	+23.75%	26.48	23.31	76.58%→81.12%
**Mechanical (Inc)**	135405	127480	−7925	−5.85%	8.08	5.41	23.43%→18.88%
**Transport (Inc)**	104926	100861	−4065	−3.87%	6.27	4.29	10.26%→9.30%
**All causes (Prev)**	187256	202052	+14796	+7.90%	11.20	8.59	n/a
**Proxy (Prev)**	102281	119503	+17222	+16.84%	6.12	5.08	54.62%→59.14%
**Falls (Prev)**	83331	101716	+18385	+22.06%	4.99	4.33	81.47%→85.12%
**Mechanical (Prev)**	18950	17788	−1162	−6.13%	1.13	0.76	18.53%→14.88%

**FIGURE 1:**
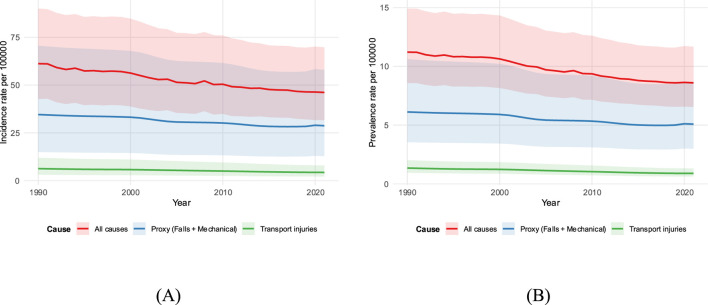
Time trends in rates, ages 20–40, both sexes (1990–2021). **(A)** Incidence; **(B)** prevalence for all causes, athletic proxy (falls + mechanical), and transport. Lines show rates per 100,000 with shaded 95% uncertainty intervals.

In contrast to increasing counts, population-weighted rates decreased significantly. Figure 1 illustrates these long-term trajectories; the all-cause incidence rate decreased from 61.18 to 46.12 per 100,000 [annual percent change (APC) −0.94%], while the athletic proxy rate decreased from 34.56 to 28.72 per 100,000 (APC −0.74%). Transport injuries showed a similar downward trend. Prevalence followed a similar pattern (Table 1), where absolute proxy cases increased by 16.84%, while the rate per 100,000 decreased.

### 3.2 Sex-specific burden

**FIGURE 2:**
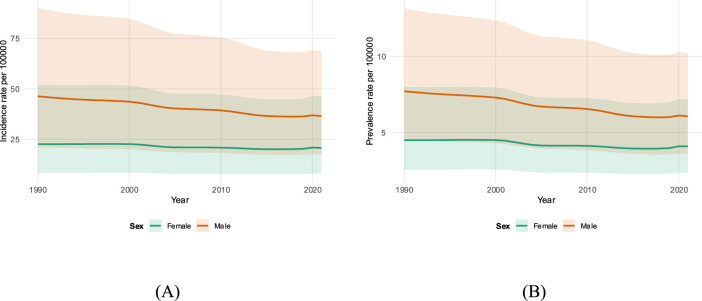
Sex-specific athletic-proxy rates, ages 20–40 (1990–2021). **(A)** Incidence; **(B)** prevalence for male and female individuals. Lines show rates per 100,000 with shaded 95% uncertainty intervals.

A persistent male excess was evident throughout the study period. As shown in Table 2, the 2021 proxy incidence rate was nearly double in male individuals (36.49 per 100,000) compared to female individuals (20.76 per 100,000). Figure 2 displays these sex-specific trends; although decreased in both sexes, male individuals had a steeper annual reduction (APC −0.88%) than female individuals (APC −0.46%).

**TABLE 2:** Sex-specific proxy rates (2021) and APCs (1990–2021).

Metric	Male	Female
Incidence rate per 100k (2021)	36.49	20.76
Incidence APC (1990–2021)	−0.88%	−0.46%
Prevalence rate per 100k (2021)	6.06	4.08
Prevalence APC (1990–2021)	−0.92%	−0.52%

### 3.3 Cause mix and age distribution

**FIGURE 3:**
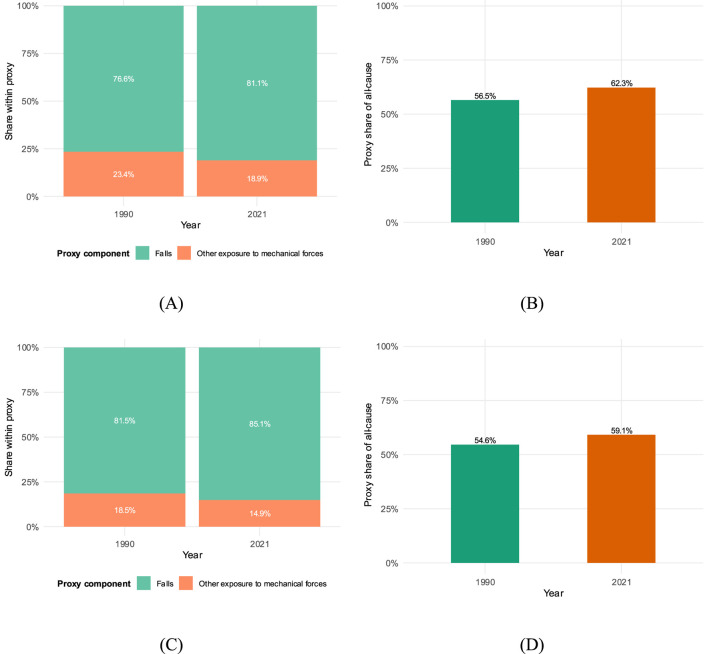
Proxy composition endpoints (1990 vs. 2021), ages 20–40, both sexes. **(A)** Incidence: falls vs. mechanical shares; **(B)** incidence: proxy vs. non-proxy share of all-cause; **(C)** prevalence: falls vs. mechanical shares; **(D)** prevalence: proxy vs. non-proxy share of all-cause. Bars are 1990 and 2021 endpoints with percentage labels.

The composition of the athletic proxy shifted over time. Figure 3 reveals that falls have become increasingly dominant. By 2021, falls accounted for 81.12% of incident proxy cases, an increase from 76.58% in 1990 (Table 3). Conversely, the share of cases attributed to “other exposure to mechanical forces” decreased.

**TABLE 3:** Proxy composition endpoints (1990 vs. 2021).

Measure	Component	1990 count	2021 count	Share (1990→2021)
Incidence	Falls	442518	547631	76.58%→81.12%
Incidence	Other mechanical	135405	127480	23.43%→18.88%
Prevalence	Falls	83331	101716	81.47%→85.12%
Prevalence	Other mechanical	18950	17788	18.53%→14.88%

**FIGURE 4:**
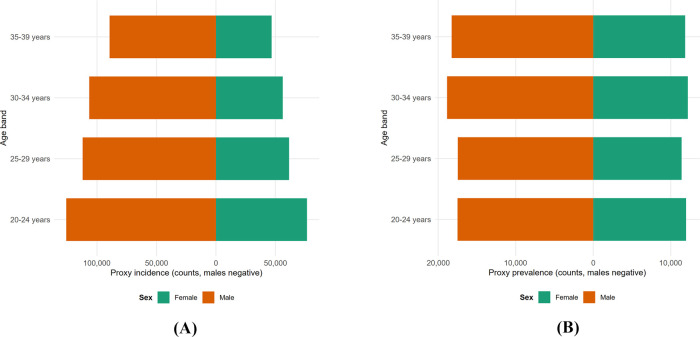
Age–sex distribution of the athletic proxy in 2021. Mirrored pyramids of **(A)** incidence and **(B)** prevalence rates per 100,000 by age band (20–24, 25–29, 30–34, and 35–39); male individuals on the left and female individuals on the right.

The burden is not evenly distributed across young adulthood. Figure 4 mirrors the age–sex distribution for 2021, showing that incidence (Panel A) and prevalence (Panel B) are the highest in the 20–24 demographic for both sexes, gradually tapering in the 35–39 age band.

### 3.4 Absolute burden and future forecasts

**FIGURE 5:**
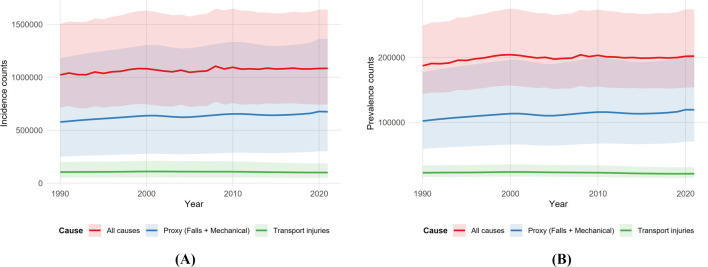
Incidence counts with 95% uncertainty intervals, ages 20–40, both sexes (1990–2021) **(A)**. Prevalence counts with 95% uncertainty intervals, ages 20–40, both sexes (1990–2021) **(B)**. All causes, athletic proxy, and transport.

Although rates have decreased, the absolute number of injuries requiring care has increased. Figure 5 visualizes this divergence, displaying the growing absolute counts for incidence and prevalence from 1990 to 2021.

**FIGURE 6:**
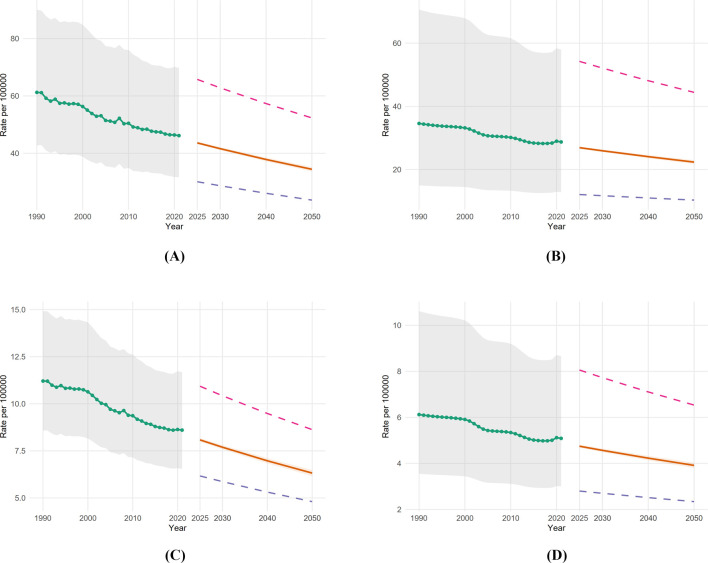
Forecast sensitivity to 2050, ages 20–40, both sexes. **(A)** Incidence: all causes; **(B)** incidence: athletic proxy; **(C)** prevalence: all causes; **(D)** prevalence: athletic proxy. Central forecasts with 95% prediction intervals plus fits to the historical lower and upper series.

**FIGURE 7:**
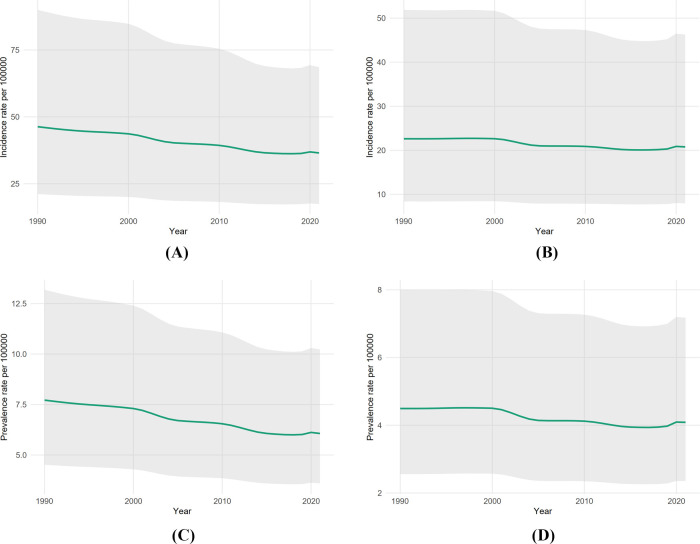
Sex-specific athletic-proxy rates with 95% uncertainty intervals, ages 20–40 (1990–2021). **(A)** Male incidence; **(B)** female incidence; **(C)** male prevalence; **(D)** female prevalence.

Looking forward, Figure 6 presents central forecasts through 2050. Assuming current trends continue, incidence rates for the athletic proxy are projected to decrease further to approximately 23 per 100,000 by 2050 (Panel B). Figure 7 provides an expanded view of the historical sex-specific rates with full 95% uncertainty intervals to support these sensitivity analyses.

## 4 Discussion

### 4.1 Principal findings

This analysis provides a focused global view of knee dislocation in young adults by isolating the portion of the burden most consistent with athletic trauma. The main findings are threefold: first, absolute incident and prevalent case counts increased between 1990 and 2021, while properly weighted rates decreased for all causes and the athletic proxy. Second, the athletic proxy accounted for the majority of the young-adult burden, with its share increasing over time, and falls dominated the proxy mix in every year. Third, a persistent male excess remained evident across the period. Trend summaries showed gradual, sustained decreases rather than abrupt inflections (Figure 1). These patterns highlight that while individual risk (rate) is decreasing, the total volume of injuries (counts) requiring healthcare resources continues to increase due to population factors.

### 4.2 Comparison with previous studies

Our findings are directionally consistent with prior global work by Chen et al. (2024), who reported decreasing age-standardized rates and a male excess in the general population using GBD 2019 data. However, our analysis refines these conclusions by isolating the 20–40 age band, revealing the “Falls” mechanism; our primary athletic proxy comprises over 80% of the burden in this demographic. This proportion is notably higher than that in general-population cohorts, where transport accidents and frailty-related falls in the elderly dilute the signal of athletic trauma.

Methodological differences likely explain the magnitude discrepancies between our GBD-based estimates and clinical reports. For instance, although we estimate a proxy incidence rate of approximately 28.72 per 100,000 in 2021, Weber et al. (2025) reported a much lower incidence of 0.44–0.54 per 100,000 in Germany (Weber et al., 2025). This variation highlights the distinction between population-level surveillance (GBD), which may capture a broader spectrum of dislocations, including spontaneous reductions and those managed non-operatively, and hospital-based registries (Becker et al., 2013; Randall et al., 2024) that typically capture only the most severe cases requiring surgical reconstruction or tertiary care. Despite these magnitude differences, the demographic patterns remain highly consistent. Our observed male-to-female incidence ratio of nearly 2:1 aligns closely with the 50%–56% male prevalence reported by Weber et al. (2025) and the young male dominance noted in the systematic review by Randall et al. (2024).

Furthermore, our classification of “Falls” as an athletic proxy is supported by mechanism-of-injury data. Moatshe et al. (2017b) identified that low-energy sports trauma (often involving twisting or planting) is frequently misclassified or generalized as falls in non-sports registries. In their cohort, these low-velocity mechanisms were a leading cause of knee dislocation, contradicting older assumptions that high-energy motor vehicle accidents are the primary cause across all regions. Our data confirm this shift on a global scale: although transport injury rates have decreased largely due to road safety improvements, the relative share of the athletic proxy has increased, suggesting that sports-related mechanisms are becoming the predominant driver of knee dislocation in young adults.

Similar trends have been observed in related joint pathologies. Jin et al. (2025) reported a global decrease in shoulder dislocation burden among adolescents and young adults from 1990 to 2021, with incidence decreasing from 107.32 to 80.22 per 100,000, predominantly in male individuals and linked to high-contact sports. In contrast, the burden of knee osteoarthritis (KOA), a known long-term sequela of knee dislocations, has increased globally (prevalence increasing to 374.7 million cases) (Chen et al., 2025). This divergence between falling incidence rates of acute injury and increasing prevalence of chronic sequelae suggests that although prevention may be improving *per capita*, the cumulative burden of past injuries and population aging continues to strain health systems.

Finally, the connection to sports is reinforced by parallel data on patellar dislocations. Dai et al. (2024) found that more than 50% of lateral patellar dislocations were sports-induced, sharing similar bone-bruise patterns and mechanisms with the knee dislocations observed in our proxy. Similarly, Lyons et al. (2024) noted increasing sports-related patellar dislocations in US emergency departments. Collectively, these comparisons suggest that the “Falls” and “Mechanical forces” trends observed in our GBD analysis accurately reflect the changing landscape of athletic trauma, in which high-velocity transport injuries are being superseded by sports-related mechanisms in the young adult population.

### 4.3 Implications and explanations

The observed decrease in rates may reflect improved prevention in organized sport, better safety standards, and advances in training and conditioning. However, the increasing absolute counts despite falling rates are consistent with global population growth and larger young-adult cohorts. The prevalence-to-incidence ratio remained stable over time, suggesting that the average duration of sequelae and the resulting disability have not changed markedly at the global level. Within the proxy, the increasing contribution of falls reinforces the practical implication that fall-prevention strategies (e.g., proprioceptive training and landing mechanics) remain critical in athletic contexts to further reduce burden.

### 4.4 Limitations and future research

This study has several limitations. First, the proxy is indirect and cannot fully exclude non-athletic falls or mechanical forces (e.g., domestic accidents), which likely biases estimates toward the null in settings where non-athletic mechanisms are common in young adults. Second, the analysis is global rather than regional, which masks heterogeneity by country, sport, and health-system context. Third, uncertainty aggregation used published lower and upper bounds rather than draw-level propagation; although this results in conservative intervals (Figures 1, 5, 7), it limits the ability to model correlation across age bands explicitly. Finally, coding practices and case ascertainment vary by place and time, and spontaneous reduction can lead to missed cases. Future research should focus on the following: 1. validating this athletic proxy against national sports-injury registries to refine the classification; 2. conducting regional breakdowns to identify specific countries where the burden is increasing; and 3. integrating population projections to provide count-based forecasts that can better guide healthcare resource allocation.

## 5 Conclusion

Young adults aged 20–40 bear a substantial global knee dislocation burden consistent with athletic mechanisms. Despite increasing counts, population-weighted rates decreased 17% from 1990 to 2021. Male individuals face nearly double the female risk; falls account for over 80% of proxy burden. The transparent proxy approach provides reproducible surveillance where sports-injury coding is unavailable, enabling trend tracking and intervention evaluation.

## Data Availability

The original contributions presented in the study are included in the article/supplementary material; further inquiries can be directed to the corresponding author.
